# Comparison of methods used to estimate coral cover in the Hawaiian Islands

**DOI:** 10.7717/peerj.954

**Published:** 2015-05-12

**Authors:** Paul L. Jokiel, Kuʻulei S. Rodgers, Eric K. Brown, Jean C. Kenyon, Greta Aeby, William R. Smith, Fred Farrell

**Affiliations:** 1 Hawaiʻi Coral Reef Assessment and Monitoring Program (CRAMP), Hawaiʻi Institute of Marine Biology, Kāneʻohe, HI, USA; 2 Kalaupapa National Historic Park, Kalaupapa, HI, USA; 3 US Fish and Wildlife Service Pacific Islands Refuges and Monuments Office, Honolulu, HI, USA

**Keywords:** Coral reefs, Coral cover, Methods comparison, Hawaii

## Abstract

Nine coral survey methods were compared at ten sites in various reef habitats with different levels of coral cover in Kāne‘ohe Bay, O’ahu, Hawaiʻi. Mean estimated coverage at the different sites ranged from less than 10% cover to greater than 90% cover. The methods evaluated include line transects, various visual and photographic belt transects, video transects and visual estimates. At each site 25 m transect lines were laid out and secured. Observers skilled in each method measured coral cover at each site. The time required to run each transect, time required to process data and time to record the results were documented. Cost of hardware and software for each method was also tabulated. Results of this investigation indicate that all of the methods used provide a good first estimate of coral cover on a reef. However, there were differences between the methods in detecting the number of coral species. For example, the classic “quadrat” method allows close examination of small and cryptic coral species that are not detected by other methods such as the “towboard” surveys. The time, effort and cost involved with each method varied widely, and the suitability of each method for answering particular research questions in various environments was evaluated. Results of this study support the finding of three other comparison method studies conducted at various geographic locations throughout the world. Thus, coral cover measured by different methods can be legitimately combined or compared in many situations. The success of a recent modeling effort based on coral cover data consisting of observations taken in Hawai‘i using the different methods supports this conclusion.

## Introduction

Over the past three decades a series of methods have been used to estimate coral cover throughout the Hawaiian Archipelago. These methods vary in the cost of equipment required, the amount of time required to conduct the survey, and the time needed to analyze images and record data. Methodologies evolved over the past 30 years due to technical advances in photography, digital video and most recently digital photography. Some methods are more appropriate for surveys over broad geographic areas while others are more suitable for intensive and detailed analyses of cover directed at coral community structure. The choice of method is very dependent on the question being asked. Some research programs only involve measures of coral cover while others involve simultaneous measurements of factors such as coral, algae, fishes, invertebrates and rugosity. In these cases, the operational compatibility of the coral census method with other assessments influences method selection. But to what extent are all of these methods comparable? Can we legitimately combine coral cover measured by various methodologies? The need for coral reef monitoring in Hawaiʻi was evaluated during a major workshop (Maragos & Grover-Dunsmore, 1999) that identified the need for methodology needed for long-term monitoring in Hawaiian waters. In response to the workshop appropriate methodology was developed by Brown et al. (2004) for the Hawaii Coral Reef Assessment Program (CRAMP), which has been monitoring Hawaiian reefs since 1999. Other programs also developed in Hawaiʻi with specific needs for estimating coral cover, so there was a need to compare results from the various measurements. The purpose of this investigation was to evaluate the comparability of transect data developed using different methodologies used in the Hawaiian Archipelago to measure coral cover.

## Materials & Methods

This project was directed at evaluating estimates of coral cover as determined from the various methods commonly used in Hawaiʻi in much the same way as fishery catch from different methods is compared (e.g., Huse, Løkkeborg & Soldal, 2000; Miller, 2013). The specific question asked was whether or not any of the coral survey methods produced consistently higher or lower estimates of coral abundance. Such differences could compromise analysis of integrated data sets from throughout Hawaiʻi developed by different observers over the past decade.

**Figure 1:**
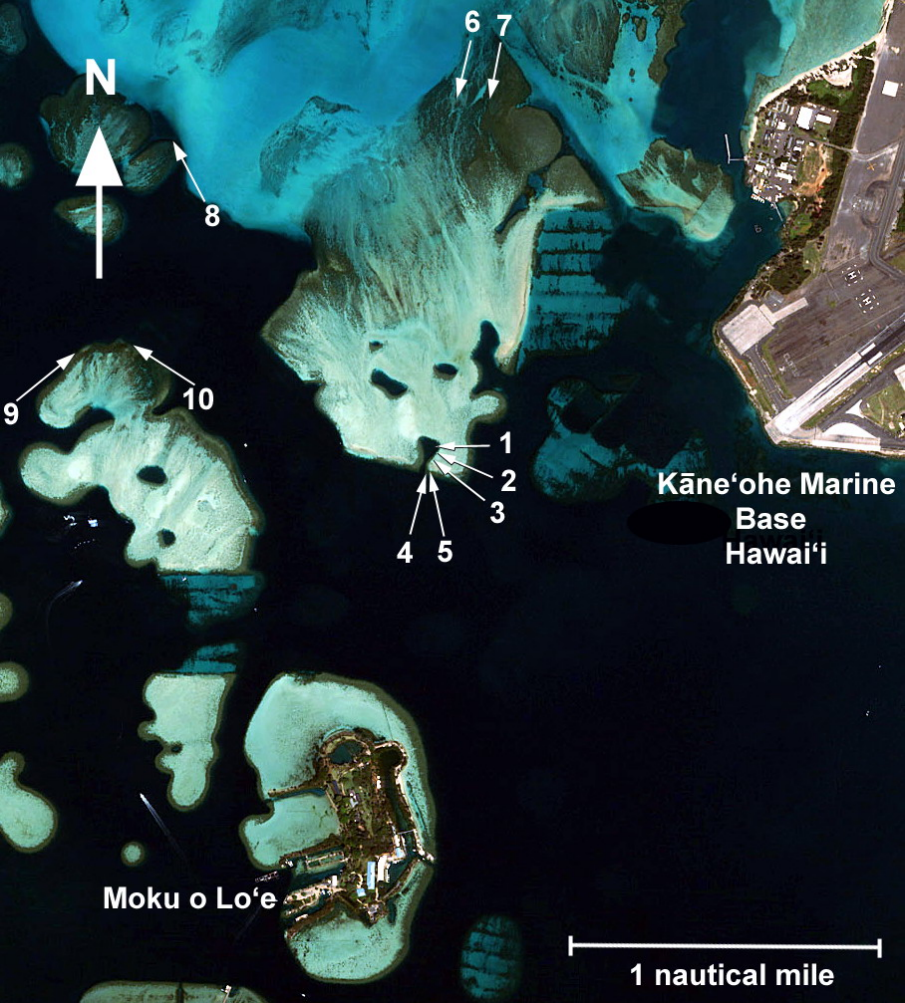
Map of sites. Aerial view of Kāne‘ohe Bay showing location of the 10 comparison sites. Quickbird Imagery Digital Globe.

Nine methods that have been widely used in the Hawaiian Archipelago were selected for comparison and applied collectively under controlled conditions. These are representative of methods that have been used previously in Hawaiʻi, although there have been minor differences resulting from variations in number of points sampled, area sampled, type of images taken or length of transect. The time required to run each transect, time required to process data and time to record the results were documented for each method. Cost for hardware and software required was also tabulated. Ten sites in various habitats having different levels of coral cover were selected in Kāneʻohe Bay for the inter-comparison experiment (Fig. 1 and Table 1). Mean estimated coverage at different sites ranged from less than 10% cover to greater than 90% cover. For purposes of this study, observers highly skilled in each particular method were used with different observers for each method. At each of the ten sites, 25 m transect lines were laid out and fastened to the bottom. Transect lines remained until each of the nine methods were tested on the deployed line. All sites were surveyed over a two-day period.

**Table 1:** Location and depth of the study sites.

Site	Depth (m)	Latitude	Longitude
1	2	21°26.587′	157°47.168′
2	6	21°26.587′	157°47.168′
3	2	21°26.587′	157°47.168′
4	1	21°26.565′	157°47.189′
5	9	21°26.565′	157°47.189′
6	2	21°25.995′	157°47.204′
7	2	21°25.995′	157°47.204′
8	4	21°26.940′	157°47.748′
9	2	21°27.634′	157°47.817′
10	7	21°26.750′	157°47.639′

The nine methods that were compared are as follows:

1. *Quadrat*
  The visual Quadrat method based on area estimates has been widely used in Hawaiʻi for the past 30 years (e.g., Maragos, 1972; Jokiel & Coles, 1974; Jokiel & Maragos, 1978; Maragos & Jokiel, 1986; Jokiel & Tyler, 1992; Jokiel, Cox & Crosby, 1993; Jokiel & Brown, 1998) and pre-dates availability of video or reliable photographic methods. Coral cover is estimated using a 1 m^2^ quadrat frame divided into 100 (10 × 10 cm) smaller squares. The observer visually estimates coral cover *in situ* and records the data on an underwater writing slate. Coral area filling one of the squares occupies 1% of the frame, so the observer uses the grid to estimate total area of each species encountered. One advantage of this method is that a transect line is not required because the frame can be flipped 25 times to measure contiguous non-overlapping areas without reference to the line. In this study, the quadrat was moved successively along the entire 25 m transect line without overlap, encompassing a total area of 25 m^2^. The coral coverage by species was estimated in each frame. During this study, PL Jokiel made the measurements using this method.
2. *Random*
  Various types of *in situ* random point sampling methods have been developed (Reed, 1980). The most widely used random point intercept method currently used in Hawaiʻi is that adopted by NOAA as part of their fish habitat utilization studies (FHUS) as described at a website at http://ccma.nos.noaa.gov/ecosystems/coralreef/hi_rfh.html. This method uses a 1 m^2^ quadrat divided into 100 (10 × 10 cm) smaller squares. Sampling points are randomly chosen intersections of the strung lines of the quadrat. Each quadrat position along the transect line is randomly chosen before the diver enters the water such that there is one randomly placed quadrat frame within every 5 m interval along the transect. If the meter mark is an odd number, then the quadrat is placed on left side of the tape; if even, it is placed on the right. Twenty-five random points per quadrat are scored. One set of random points are selected prior to fieldwork and used for all frames. The cover below each of the 25 random intercept points is scored as if looking at the quadrat in a two dimensional plane (i.e., as in a photograph). Percent cover values are calculated by dividing the number of recorded points by the number of selected intersections. KS Rodgers made measurements using this method during this study.
3. *Point Intercept Transect (PIT)*
  The Point Intercept transect (PIT) is described in Hill & Wilkinson (2004) and Obura (2014). The PIT has been used in Hawaiʻi in a coral disease monitoring program (Aeby et al., 2011) and was included in this study. Data on coral species under each point located at 50 cm intervals along a 25 m transect is recorded, yielding a total of 51 points per transect. Data were taken along two 25 m transect lines spaced end to end with a five meter gap. Percent coral cover is determined by dividing the number of points recorded as live coral by the total number of points and multiplying by 100. A more widely used technique called the line intercept transect (LIT) is essentially the same but measures are taken at a higher resolution of 1 cm (Obura, 2014). To our knowledge, the classic LIT has not been used in Hawaiʻi. Dollar (1982) used a line transect method based on the methods of Loya (1972) and Porter (1972). For each transect, a 45 m cord was stretched over the reef and a chain draped along the transect line. The number of links lying over each coral colony or non-coral substratum was recorded. Use of chain links to count cover adds a vertical dimension and will give a higher coral cover than planar methods. During this study G Aeby made the measurements using the PIT method.
4. *CRAMP RAT*
  The Hawaiʻi Coral Reef Assessment and Monitoring Program (CRAMP) Rapid Assessment Technique (RAT) is a highly abbreviated version of the CRAMP monitoring protocol, consisting of a single fish transect, a single benthic transect, a rugosity measurement, a sediment sample and various qualitative habitat observations (Jokiel et al., 2004; Rodgers et al., 2015). The RAT is designed to produce quantitative spatial data that is consistent with and comparable to data taken at the permanent monitoring sites. The power of the RAT lies in large numbers of replicates taken over the spatial range of a given habitat. The assessment program expands the ability to describe habitats and spatial distributions of Hawaiian reef organisms in relation to various environmental factors. However, the assessment protocol requires a small fraction of the human effort and cost per site in comparison to the monitoring sites. Assessment data can be used with monitoring data for spatial comparisons.
  Benthic cover is determined using high resolution digital images taken along a 10 m transect using an Olympus 5050 zoom digital camera with an Olympus PT015 underwater housing. The camera is mounted to an aluminum monopod frame, 1.7 m from the substrate to provide a 50 cm × 69 cm image. The 20 non-overlapping images from each 10 m transect are imported into an ecological analysis program PhotoGrid where 50 randomly selected points are projected onto each image for a total of 1,000 points per transect. Percent cover, species count and diversity of corals, algal functional groups and substrate cover are quantified.
  Precision using photographic techniques was determined to be high (∼95% similarity among observers) compared to *in situ* observations (Brown et al., 2004). Although initial costs are high, cost effectiveness surpasses visual techniques after only ten surveys (Brown et al., 2004). While post-processing time is involved, costly underwater dive time is greatly reduced with the use of this technique. The method allows for archiving and data verification, which is critical in addressing further questions, and in quality control. Recent technological advances in digital still cameras have alleviated the disadvantage of limited image resolution. KS Rodgers made measurements using this method.
5. *Video transect*
  A digital video camera in an underwater housing is used to record the substrate along two 25 m transects placed end to end with a 5 m separation. A red filter in the housing is used over the lens at depths greater than ∼4 m. The videographer swims approximately 1 m above the transect line with the camera lens pointing directly downward, and additionally records 360° views of the surrounding area at the beginning and end of each transect line. The software program DVRaptor-RT Video (Canopus Corporation, Kobe, Japan) is used to capture adjacent, non-overlapping still frames along the length of each transect line. Images are enhanced in ACDSee™ (ACD Systems, Seattle, Washington, USA) when needed to improve the quality of the images. The number of captured frames varied among the 20 transects at the 10 sites (range 29–36 frames/transect) in the present study due to variation in the height of the videographer (KS Rodgers) above the transect lines. Accordingly, the number of captured still frames randomly selected for analysis varied (range 16–25) so as to include 16 m^2^/transect benthic area (Kenyon et al., 2006a). J Kenyon analyzed selected frames for coral cover by species according to the method described in Kenyon et al. (2005).
6. *Towed-diver*
  Towed-diver surveys have been widely used throughout Hawaiʻi by the Coral Reef Ecosystem Division (CRED) of NOAA’s Pacific Islands Fisheries Science Center following the methods of Kenyon et al. (2005). Habitat digital still frames sampled from videotapes at 30 s intervals as the towboard moves over the bottom at ∼0.8 m/s are analyzed for coral percent cover using the methods of Kenyon et al. (2005). In the present study, three photos were taken with an Olympus 5050 digital camera in an Olympus PT015 underwater housing to simulate images produced by the towed-diver method. One image was taken at the beginning and end of the first 25 m transect and one at the end of the second 25 m transect (i.e., three images over 50 m). This spacing and image area (∼1 m^2^) is equivalent to that produced by the towed-diver method (Kenyon et al., 2006a). The field survey was supervised on site by J Kenyon and used her equipment. The processing of the images was carried out entirely by J Kenyon.
7. *Photoquad (Photographic Transect)*
  An Olympus 5050 digital camera with a wide-angle lens and an Olympus PT015 underwater housing was used to obtain the images. The camera was positioned above a 1 m^2^ quadrat strung into 100 subdivisions at the proper distance needed to include the entire frame in the photograph. Images were taken along the entire 25 m transect line without overlap. A total of 25 m^2^ was used to visually determine the percent substrate cover. The software program ACDSee™ 32 Browser is used for image viewing with data input directly into Microsoft Excel. The amount of coverage of each substrate type is estimated in each subsquare, and then summed to determine the total cover for each species in a manner similar to the *in-situ* quadrat method. Coral cover is recorded by species. During this study the quadrats were photographed by F Farrell with P Jokiel moving the frame over the transect line. The laboratory analysis of the images was conducted by K Uchino.
8. *Estimate*
  In this method a visual *in situ* estimate of percent coral cover by species is visually recorded by a diver swimming along a transect 25 m in length and 5 m in width. The method requires an experienced observer. During this study KS Rodgers made the measurements using this method.
9. *NOAA ground truth*
  NOAA’s National Ocean Service Biogeography Team uses this method for field validation and accuracy assessment of benthic habitat maps (NOAA, 2005; Monaco et al., 2005). The location of a sampling station for map accuracy assessment is determined using a stratified random sampling regime as per Congalton (1988), while stations used for field validation are selected based on specific features in the imagery, which are difficult to identify without further fieldwork. The stations are located using navigational GPS and marked with a temporary buoy. A 7 m radius around the marker delineates the area surveyed. Geomorphologic structure and biological cover based on the NOAA classification scheme are recorded (Coyne et al., 2003). Coral cover is estimated in 10% increments, which are subsequently placed into one of the following four categories: <10%, 10%–49%, 50%–89%, and 90%–100%. Thus, the estimated total coral cover may fall into only one of four categories, which might be termed “none,” “low,” “medium” and “high.” This method requires a skilled observer, but takes very little time. For shallow sampling stations, the observer will often view the bottom through a “look box” from a boat or kayak without actually getting into the water. Deeper sampling stations or those with poor visibility are assessed by snorkel. Digital photographs of stations sampled by snorkeling are often collected. WR Smith made measurements for this investigation.

### Statistical methods

Number of species was examined using a General Linear Model (GLM) one-way ANOVA with number of species as the dependent variable (*N* = 10 sites) and nine methods (Quadrat, Random, Point Intercept, CRAMP RAT, Video, Towed-diver, Photoquad, Estimate, and NOAA Ground Truth) as the factor. Transects at each of the 10 sites were averaged for methods using two 25 m transects (Point Intercept, video, towed-diver).

Comparing both coral cover and species count data among methods utilized a one-way ANOVA. Percent coral cover was arcsin-square root transformed to meet the assumptions of normality and homogeneity of variances. Post-hoc multiple comparisons among means were conducted using a Tukey HSD test when ANOVA results detected significant differences among methods.

A ranking test was applied to the site data in order to determine whether or not any of the methods produced estimates that were consistently higher or consistently lower than the others. For each site, each method was ranked with a rank of 1 given to the method yielding the highest coral cover for the site and a rank of 9 to the method that gave the lowest coral cover for the site. Thus, a method that yielded the highest coral cover at all 10 sites would have an average rank of 1. A method that yielded the lowest coral cover at all 10 sites would yield a rank of 9. If all methods yielded the exact same number at each site then all methods would have a rank of 5. The rank data can then be analyzed statistically to determine if there was a consistent difference in the estimate of coral cover produced by any of the various methods.

Coral cover for each method was ranked from highest to lowest at each site. A one-way ANOVA was then conducted with untransformed ranks as the dependent variable and method as the independent factor. This procedure allowed a more appropriate comparison among the methods by separating out the effect of habitat.

The coral cover variance structure among methods was plotted to display which method generated the most variable data set and consequently the lowest statistical power to detect spatial differences.

## Results and Discussion

**Figure 2:**
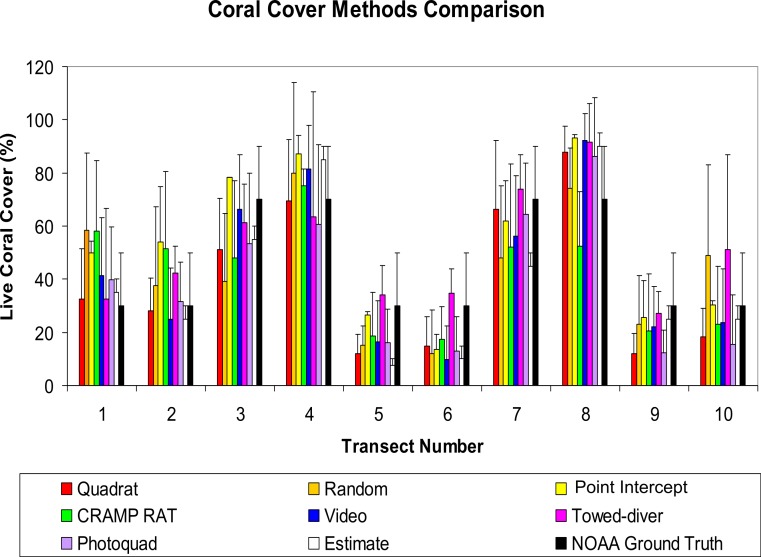
Error bars for the first 7 methods (rainbow colors) are Standard Deviations. Error bars for the last two methods (white for Estimate and black for NOAA Ground Truth) represent estimated range.

A comparison of the costs and time required for each method is presented as Table 2. The initial equipment cost varies by three orders of magnitude among methods. For example, the visual “Estimate” method takes one person several minutes and costs practically nothing while a “Towed-diver” survey requires two divers, two tow-boards, cameras, communication gear and a towing boat and crew. Considerable post-processing of the imagery is required for the video transect. A summary of the total coral cover data for each method at each site is shown as Fig. 2. Note the high variance of all methods due to the inherent “patchiness” of reef coral communities. Coverage by species for each method is shown in Table 3. A summary of the number of coral species recorded by each method is shown in Table 4. The values for coral cover are for a single site (*n* = 1) (Table 5). Sub-samples are not independent estimates, so the error bars are only useful for showing the high variance encountered in a coral reef environment. Running the same method over the same transect a number of times will give a highly variable result (e.g., see Brown et al., 2004).

**Table 2:** Summary table describing main features and costs for each method.

Method	Equipment	Data record	Transect length	Number of samples per site	Points per sample	Approx. area (m^2^)	Survey time (min)	Lab analysis (min)	Spread sheet data entry (min)	Total time (min)	Equipment cost (US Dollars)
Quadrat	1 m^2^ quadrat, writing slate	*in situ*	25 m	25	100	25	20	0	5	25	$20
Random	transect line 1 m^2^ quadrat, writing slate	*in situ*	25 m	5	25	5	15	15	1	31	$20
Point Intercept (PIT)	transect line, writing slate	*in situ*	two 25 m lines	2	51	50	20	0	5	25	$20
CRAMP RAT	transect line digital camera, PhotoGrid Software	Lab. Computer System	10 m	20	50	20	15	40	10	65	$700
Video	transect line video camera, D/V Raptor, CPCe Software	Lab. Computer System	two 25 m lines	34–47	50	50	5	65	5	75	$1,500
Towed-diver	digital camera, Sigma Scan software, tow-board system.	Lab. Computer System	approx. one image per 25 m	3	50	3	3	5	5	13	$2,500
Photo-quad	transect line 1 m^2^ photoquad, camera	Lab. Computer System or Projector	25 m	25	100	25	15	35	5	55	$700
Estimate	transect line writing slate	*in situ*	25 m	1	estimated range	25	3	0	1	4	$2
NOAA Ground Truth	writing slate	*in situ*	7 m radius	1	estimated range	154	3	0	1	4	$2

**Table 3:** Coverage by species for each method at each site.

	Quadrat	Random	Point Intercept (PIT)	CRAMP RAT	Video	Towed-diver	Photoquad	Estimate	Ground truth	Quadrat	Random	Point Intercept (PIT)	CRAMP RAT	Video	Towed-diver	Photoquad	Estimate	Ground truth
*Montipora capitata*										*Pocillopora meandrina*								
1	15	4	25.5	30.7	20.1	22.8	20	20.0	15.0									
2	21	15	17.6	30.6	9.4	8.8	23	10.0	18.0									
3	37	22	53.9	27.4	44.1	42.7	39	25.0	35.0									
4	29	54	39.2	33.7	37.6	20.1	30	40.0	42.0					0.2				
5	9.9	15	21.6	16.8	14.7	34.9	12	3.5	21.0									
6	8.1		8.8	1.4	6.7	15.3	7	4.0	15.0					0.1		0.02		
7	27	26	18.6	40.7	21.1	3.4	44	10.0	7.0		1.6							
8	2.3	4	5.9	2.1	8.9	20.1	6.5	25.0	56.0	0.2						0.3		
9	8.1	14	16.7	14.2	16.2	16.7	9	9.5	15.0							0.1		
10	8	33	7.8	16.1	10.4	42.5	11	15.0	21.0									
*Porites compressa*										*Pavona varians*								
1	18	14	24.5	27.5	21.2	9.9	20	15.0	15.0									
2	16	22	36.3	20.9	15.5	33.7	8.4	15.0	12.0									
3	14	17	24.5	20.9	22.4	18.7	14.2	30.0	35.0									
4	41	26	48.0	41.4	43.8	43.4	30.4	45.0	28.0									
5	1.8		3.9	1.8	1.6		3.8	4.0	9.0									
6	1.1	2.4	1.0	1.5	0.2	0.7	0.7	2.0	9.0									
7	39	21	41.2	10.4	34.0	70.5	20.6	25.0	63.0									
8	84	68	85.3	48.8	83.3	68.8	79.4	65.0	14.0	1	1.6		1.1					
9	3.5	7.2	7.8	5.2	4.9	9.1	2.9	15.0	15.0	0.02								
10	10	16	21.6	7.0	13.3	8.8	4.9	10.0	9.0									
*Porites lobata*										*Fungia scutaria*								
1					0.1													
2																		
3																		
4																		
5							0.7											
6	5.2	8.8	2.9	3.8	2.8	1.3	5.2	4.0	6.0									
7	0.1		1.0	0.1	0.7		0.3	9.0										
8			2.0															
9										0.1								
10	0.1																	
*Pocillopora damicornis*										*Montipora patula*								
1	0.03																	
2	0.02																	
3	0.02			0.1														
4							0.1											
5	0.2		1.0	0.1	0.1		0.1											
6	0.2	0.8	1.0		0.1		0.3											
7	0.1		1.0	0.2	0.3		0.1	1.0										
8	0.1			0.2	0.1				0.2	0.8		0.1		2.8				
9	0.3	1.6	1.0	0.1	0.7	1.4	0.4	0.5	0.4				0.3					
10			1.0				0.1											

**Table 4:** Number of coral species recorded per site by each method.

Species recorded per transect									
Site no.	Quadrat	Random	Point Intercept (PIT)	CRAMP RAT	Video	Towed-diver	Photoquad	Estimate	NOAA ground truth
1	3	2	2	2	3	2	2	2	2
2	3	2	2	2	2	2	2	2	2
3	2	2	2	3	2	2	2	2	2
4	2	2	2	2	2	2	3	2	2
5	3	1	3	3	3	1	4	2	2
6	4	3	4	3	5	3	5	3	3
7	4	3	4	4	4	2	4	4	2
8	5	4	3	5	3	3	3	2	2
9	6	2	2	3	4	3	3	2	2
10	3	2	3	2	3	2	3	2	2
**Mean**	**3.5**	**2.3**	**2.7**	**2.9**	**3.1**	**2.2**	**3.1**	**2.3**	**2.1**
S.D.	1.2	0.8	0.8	0.9	0.9	0.6	0.9	0.6	0.3

**Table 5:** Mean rank scores and mean % coral cover (mean of 10 sites) for each method.

Transect	Quadrat	Rank	Random	Rank	Point intercept (PIT)	Rank	CRAMP RAT	Rank	Video	Rank	Towed-diver	Rank	Photoquad	Rank	Estimate	Rank	NOAA Ground Truth	Rank
**1**	32.6	**7**	17.7	**9**	50.0	**2.0**	58.2	**1.0**	41.4	**3.0**	32.7	**6.0**	39.8	**4.0**	35.0	**5.0**	30.0	**8**
**2**	37.0	**5**	37.6	**4**	53.9	**1.0**	51.5	**2.0**	24.9	**9.0**	42.4	**3.0**	31.6	**6.0**	25.0	**8.0**	30.0	**7**
**3**	51.1	**7**	39.2	**9**	78.4	**1.0**	48.4	**8.0**	66.5	**3.0**	61.3	**4.0**	53.3	**6.0**	55.0	**5.0**	70.0	**2**
**4**	69.6	**7**	80.0	**4**	87.2	**1.0**	75.1	**5.0**	81.6	**3.0**	63.5	**8.0**	60.8	**9.0**	85.0	**2.0**	70.0	**6**
**5**	11.9	**8**	15.2	**7**	26.5	**3.0**	18.7	**4.0**	16.4	**5.0**	34.9	**1.0**	16.1	**6.0**	7.5	**9.0**	30.0	**2**
**6**	14.6	**3**	12.0	**6**	13.7	**4.0**	6.7	**9.0**	9.8	**8.0**	17.3	**2.0**	13.2	**5.0**	10.0	**7.0**	30.0	**1**
**7**	66.4	**3**	48.0	**8**	61.8	**5.0**	51.5	**7.0**	56.1	**6.0**	73.9	**1.0**	64.5	**4.0**	45.0	**9.0**	70.0	**2**
**8**	87.8	**5**	74.4	**7**	93.2	**1.0**	52.3	**9.0**	92.4	**2.0**	91.7	**3.0**	86.2	**6.0**	90.0	**4.0**	70.0	**8**
**9**	12.4	**9**	23.2	**5**	25.5	**3.0**	19.5	**7.0**	22.0	**6.0**	27.2	**2.0**	12.4	**8.0**	25.0	**4.0**	30.0	**1**
**10**	18.2	**8**	48.8	**2**	30.4	**3.0**	23.1	**7.0**	23.6	**5.0**	51.2	**1.0**	15.5	**9.0**	25.0	**6.0**	30.0	**4**
**Sum**	401.6	**62.0**	396.1	**61.0**	520.6	**24.0**	405.0	**59.0**	434.7	**50.0**	496.1	**31.0**	393.4	**63.0**	402.5	**59.0**	460.0	**41.0**
**Mean**	40.2	**6.2**	39.6	**6.1**	52.1	**2.4**	40.5	**5.9**	43.5	**5.0**	49.6	**3.1**	39.3	**6.3**	40.3	**5.9**	46.0	**4.1**
**SD**	27.3	**2.1**	23.8	**2.3**	27.9	**1.4**	21.9	**2.8**	29.1	**2.3**	23.1	**2.3**	26.0	**1.8**	28.7	**2.3**	20.7	**2.9**

### Outcome of statistical analyses

**Figure 3:**
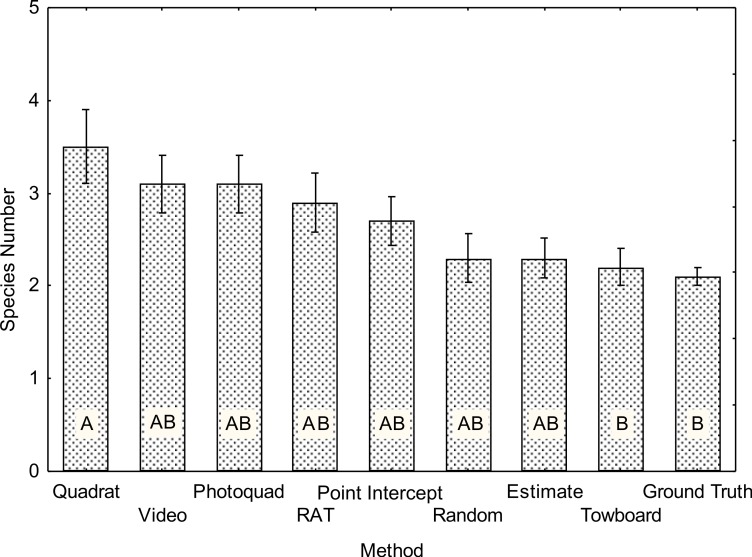
Average species count for each of the benthic survey methods (*N* = 10). Mean number ± 1 SE. The same letter denotes homogeneous means using a Tukey HSD post-hoc test on square root transformed data (*F*_8,81_ = 3.2 *p* = 0.003).

Measured species count (Table 4 and Fig. 3) differed significantly among methods (*F*_8,81_ = 3.2, *p* = 0.003). The “Quadrat” method detected the highest number of species while the “NOAA Ground Truth” and “Towed-diver” methods showed the lowest number of species. The “Quadrat” method detected a significantly greater number of species (Fig. 3) than the other methods for a number of reasons. In the first place, this method surveys every coral within the 25 m^2^ area. Methods that involve sampling a limited number of points generally will miss rare species. The “Quadrat” method involves close and direct observation of the corals by a diver who can easily detect corals such as *Pavona varians* and *Fungia scutaria* that typically grow in crevices and shaded areas that often do not show on image analysis. Close inspection by the diver allowed easy recognition of *Montipora patula*, which often cannot be differentiated from *Montipora capitata* in images. The Quadrat method requires an experienced coral reef ecologist as observer, but clearly will produce a more complete estimate of species number for a given belt transect. The “Ground Truth” and “Towed-diver” methods require viewing from a greater distance and were significantly lower in their ability to detect small, uncommon or cryptic species (Fig. 3), while the remaining six methods showed no significant differences.

**Figure 4:**
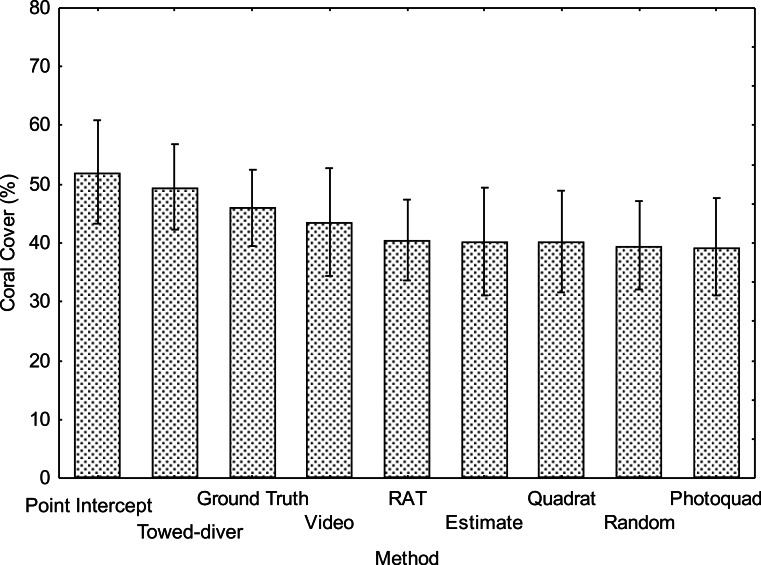
Mean coral cover for the 10 sites as measured by each of 9 methods (±S.D.) Mean number ± 1SE. No significant differences among methods were detected using a Tukey HSD post-hoc test on arcsin-square root transformed data (*F*_8,81_ = 0.38 *p* = 0.93).

**Figure 5:**
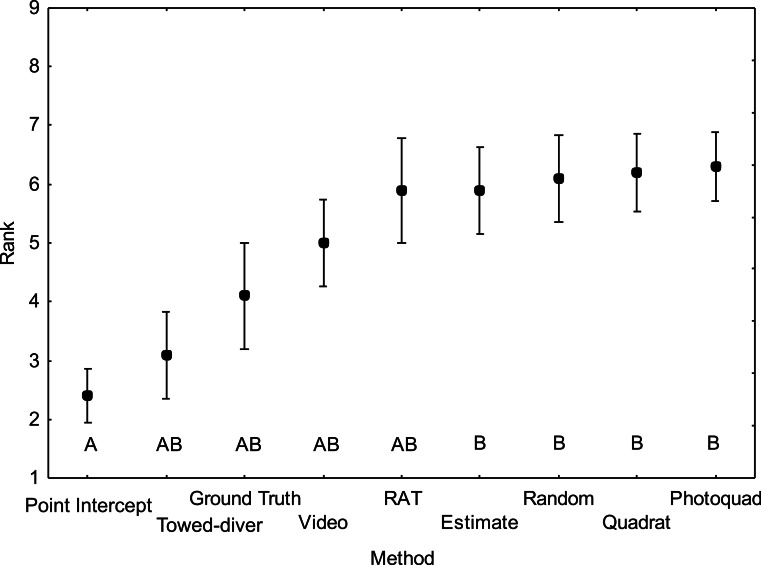
Average ranking of coral cover at each site for the nine benthic survey methods (*N* = 10) plotted from Table 5 data. Mean number ± 1 SE. Methods in each group are statistically different than those in other groups while group AB has means statistically similar to those in both groups A and B (Tukey HSD post-hoc test on raw data (*F*_8,81_ = 4.0 *p* < 0.001).

Overall mean coral cover measured at the 10 sites (Fig. 4) was not significantly different among methods (*F*_8,81_ = 0.38 *p* = 0.93) due to the inherent variance in “patchy” coral reef communities. Nevertheless, the ranking analysis (Fig. 5) detected a significant difference among methods in terms of a consistent coral cover pattern (*F*_8,81_ = 4.0 *p* < 0.001). The “Point Intercept” method typically ranked higher (lower numbered rank) at each site followed by the “Towed-diver,” “NOAA Ground Truth,” “Video,” “RAT,” “Estimate,” “Random,” “Quadrat,” and “Photoquad” method. The “Point Intercept” method probably gives a slightly higher cover estimate because wave action tends to move the transect line slightly until it catches on a coral. All points measured by the method are taken directly under the transect line, so the ranking score was significantly higher. The “Point Intercept” method was statistically similar to the “Towed-diver,” “NOAA Ground truth,” “Video,” and “RAT” methods. The “Estimate,” “Random,” “Quadrat,” and “Photoquadrat” methods, however, grouped together and ranked statistically lower at each of the ten sites. Note that there is considerable overlap between the groups A and B (Fig. 5), so distinctions are not clear statistically.

**Figure 6:**
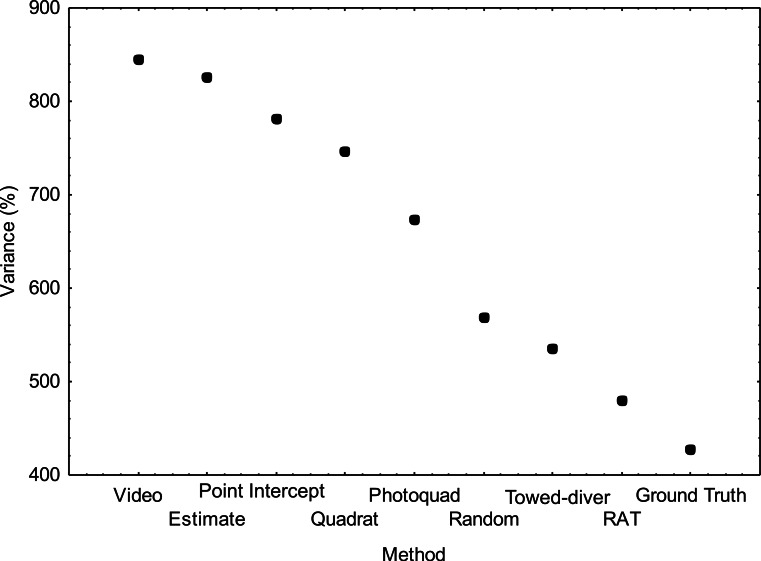
Variance (%) for each of the benthic survey methods (*N* = 10).

There is a growing recognition of the importance of power analysis and sample size calculation for the proper design of monitoring and assessment programs. Statistical power of an analysis is a function of sample variance and sample number. Thus, variance estimates determined in this comparison study are useful in determining sample size needed using different methods and thereby can help in the design of future surveys. The variance structure among methods (Fig. 6) showed that the “Video” method had the highest variance for the ten sites and therefore, lowest statistical power to detect differences among sites. In contrast, the “NOAA Ground Truth” protocol had half the variance among the sites/transects. However, this difference is most likely an artifact due to the limited number of coral cover categories used in this method. The “RAT” method used high resolution digital still images, but otherwise was similar to the “Video” approach in terms of image analysis and substrate categorization. The “Video” technique covers a larger area (50 m) with 234 to 247 image subsamples within this distance. In contrast, the RAT covers a shorter distance (10 m) and covers the entire area sampled, using 20 non-overlapping images. Thus, the higher variability in the “Video” method is likely due to the larger area being subsampled. In addition, the lower image resolution for the “Video” method may also be a factor in producing higher variability in the estimate.

Investigators who initially developed and implemented each method apparently had an “intuitive” grasp of how to develop a reasonable scheme that met their specific survey needs. All of the methods yield comparable estimates for coral cover. Each method has strengths and weaknesses and no one method will fit all research needs. The methods evaluated here were originally developed in response to the needs of specific investigations. Factors such as differences in question being asked (intensive or extensive surveys), constraints on field time or laboratory processing time, costs of hardware and software required, level of field experience of observers, utility of sampling regimes under various field conditions, need to archive images for future use, etc. led to development of diverse methods. These methods were widely used in the past and undoubtedly will continue to be used in the future, so it is of great value to know that all of them yield similar coral cover estimates.

Franklin, Jokiel & Donahue (2013) compiled a benthic cover database from the various scientific research and monitoring programs that included 37,710 total benthic cover observations from 2000 to 2009 around the main Hawaiian Islands. Coverage for each species was plotted throughout the Hawaiian Islands. This data was used to successfully develop species distribution models for dominant Hawaiian coral species, which was then tested with survey data. The excellent agreement between field observations and modeling results of the Franklin, Jokiel & Donahue (2013) report adds additional evidence that results of the different methods produce comparable coral cover estimates.

Coral cover data inevitably show very high variance due to inherent “patchiness” of reef coral distributions, so even repeat sampling of the same transect with the same method will produce quite variable results. From a statistical point of view, it appears that no one method was sufficiently different from the other methods based on the various characteristics evaluated in this study (Table 6). For example, some methods (e.g., Point Intercept) documented high values in one area (e.g., coral cover rank), but generated moderate values for other characteristics (e.g., species count). Other protocols (e.g., Estimate) spanned the range of category values, but did not represent the extreme for any one particular parameter. Finally, methods such as “Random” recorded moderate values for all of the characteristics.

**Table 6:** Summary of categorical groupings from data of coral cover methods based on species count, coral cover ranking, and variance.

Method	Species count^a^	Coral cover rank^b^	Variance^c^
CRAMP RAT	Medium	Medium	Low
Estimate	Medium	Low	High
NOAA Ground Truth	Low	Medium	Low
Photoquad	Medium	Low	Medium
Point Intercept (PIT)	Medium	High	Medium
Quadrat	High	Low	Medium
Random	Medium	Low	Medium
Towed-diver	Low	Medium	Medium
Video	Medium	Medium	High

**Notes.**

a Based on groupings of means in multiple comparison test.

b Based on groupings of means in multiple comparison test.

c Based on the following categories (high > 800, medium 500–800, low < 500).

Ideally, the optimal method would document higher species count for biodiversity considerations, rank in the middle in terms of coral cover for good precision, and have low variance for high statistical power. Perhaps the closest method is the CRAMP RAT, but only for the variables we measured. However, how well does this protocol really characterize the actual coral community compared to the other methods? Answering this question would require knowing the entire population under consideration. Therefore, any of these methods appear to be adequate for relative comparisons both spatially and temporally and the method should be selected based on other considerations.

Results of this investigation indicate that all of the methods used in the comparison are appropriate for measuring coral cover to provide a good first estimate for the general characterization of coral cover on a reef. This inter-comparison did not detect any consistent differences in the estimate provided by each method.

Numerous visual surveys of coral cover have been conducted in the past, but the information obtained has been used sparingly in the literature. In some situations, visual estimates can be more reproducible and more accurate than random-point sampling (Dethier et al., 1993). However, there is a general reluctance to place reliance on subjective visual assessments, although Kenchington (1978) indicated that it should be possible to develop training procedures and scales of assessment which enable valid comparisons to be made of coral cover in space and time. Results of this investigation support the view of Kenchington (1978) in that observers with extensive experience in quantitative assessments of coral cover produced visual estimates that were similar to results of techniques that require much more effort. Even the “NOAA Ground Truth” method, which is restricted to scoring on a scale of four divisions ranked well against the other methods.

The classic “Quadrat” method has been in use in Hawaiʻi for nearly 40 years and is simple and inexpensive to use but requires a highly experienced observer. This method is especially attractive because of the large sample size (25 m^2^, each with 100 squares = 2,500 sampling units) compared to other methods. The proximity of the observer to the substratum and the large sample area yields a much better estimate for small or rare coral species. The ability of the “Quadrat” method to detect species allows coral abundance to be classified objectively (Jokiel & Maragos, 1978) according to a system such as dominant (>10% cover), abundant (<1.0 to 10% cover), “common” (<0.1–1.0% cover), or “rare” (<0.1% cover). “Very rare” species can be defined as species not occurring in any quantitative sample, but noted in the area.

The use of towed-diver surveys was inaugurated in 1990 to assess those benthic variables considered to be important to lobster habitat on three emergent banks in the Northwestern Hawaiian Islands (Parrish & Polovina, 1994). Since that time the method has evolved under the guidance of the Coral Reef Ecosystem Division at NOAA’s Pacific Islands Fisheries Science Center in response to advances in videographic and georeferencing technology and has been adapted for mesoscale assessment of coral reef benthic habitats throughout the insular US Pacific. A primary strength of towed-diver surveys is their ability to assess the major benthic components and condition of reef habitats over spatial scales substantially greater than can be observed and documented by free-swimming divers. A typical towed-diver survey covers up to 3 km, and 6 surveys can usually be conducted each field day. The method is particularly useful for assessing remote areas that can only be visited infrequently and for short durations, where more limited sampling by free-swimming divers may not adequately characterize the diversity of habitats. Because the divers are towed underwater by a surface boat, they are able to work in sea conditions of swell or surge that are too dangerous for free-swimming divers, and can thus provide data for habitats such as windward exposures that are otherwise rarely accessible (e.g., Kenyon et al., 2005; Kenyon et al., 2006a; Kenyon et al., 2006b). They provide a permanent visual record that is amenable to re-sampling. Because the imagery is linked to geographical position by way of a GPS receiver onboard the tow boat, a survey can be sub-sampled for any spatial interval that may be of interest. A primary limitation of interpreting visual information from a towed camera is the loss of taxonomic resolution, as is seen in reduced species count in the present study relative to most other techniques (Table 4, Fig. 3). Field equipment as well as computer equipment needed to analyze imagery is expensive (Table 2), realistically limiting this method to programs with large budgets. Field and computer personnel require special training, the former to ensure safety and accuracy, the latter to ensure consistency and reproducibility.

The “Video” method evaluated in the present study was developed for use in benthic assessment surveys in the Northwestern Hawaiian Islands by a single diver tasked with estimating multiple parameters relevant to coral communities during one dive per site per year. The short field survey time required compared to most other methods in the present study (Table 2) is well suited to maximizing the time available to assess other coral parameters including site species count, colony abundance and density, and size class distributions. It can also be implemented by an experienced diving assistant without expertise in the identification of marine organisms. The permanent visual record allows comparison between observers at a later date. However, as seen in the present study, efficiency of field data collection is offset by substantial post-processing time (Table 2). Though species count in the present study was congruent with other methods (Table 4, Fig. 3), the “Video” method is not as adept at reporting small, cryptic, or encrusting taxa (e.g., *Pavona varians, Cyphastrea, Leptastrea*) when they occur as are most *in situ* methods.

Other workers in Hawaiʻi have used techniques that are similar, but not identical to those evaluated in this investigation. Dimensions of photo-transects less than the 1 m^2^ used in this investigation have been adopted by some investigators in order to accommodate the image taken by 35 mm cameras. Coles (1984) used a fixed photoquadrat measuring 1 m × 0.66 m marked by fixed galvanized iron bolts, which serve as a platform for a camera frame. Images taken with a Nikonos camera having a 28 mm wide angle lens at a focal distance of four feet correspond to the field of view of 0.66 m^2^ for each quadrat. Percent coverage of each species on each quadrat are determined by the point intercept method using 485 points superimposed on the projected image. Likewise, Dollar & Grigg (2004) used a 1 m × 0.66 m^2^ image area to accommodate the field of a wide angle lens, but used a visual estimate based on a grid of 100 subdivisions similar to that used in this study. Other variations in the different methods have been employed, but generally methods used previously are encompassed by the type of measurements evaluated in this investigation.

This study focused on techniques used in Hawaiʻi. Other studies have compared techniques not widely employed here. These include comparison of video line-intercept (Carleton & Done, 1995) or chain method (Rogers, 1999) with video, photographic, and estimated cover. The major conclusion of these studies is techniques that involve a three dimensional component (video line-intercept, chain method) will give a different estimate than methods that measure in a planar view. All of the techniques used commonly in Hawaiʻi report a planar area for coral coverage including the *in situ* line intercept method. Relatively few coral species occur in Hawaiʻi. Low coral diversity might have contributed to the agreement in results among different methods in this study. Similar evaluations in highly diverse reef systems might show greater differences between the techniques. Further, gorgonians and *Acropora* thickets are not found on shallow Hawaiian reefs, so investigators here do not have to deal with a large vertical dimension.

### Applications to monitoring

The present investigation compared methods suitable for initial site characterization along a single transect. Comparison and evaluation of monitoring techniques designed to detect subtle changes in coral cover over time is a far more complex issue. The choice of sampling design for a reef monitoring project is determined principally by the question to be answered and the sampling accuracy and precision required (Green & Smith, 1997). Such studies require rigorous sampling design and analysis in order to produce the statistical power needed for the experimental design. Statistical power is the probability of rejecting the null hypothesis or the confidence in accepting null hypotheses of no change over time for key parameters such as coral percent cover (Green & Smith, 1997). The development and testing of the monitoring design employed by CRAMP has already been discussed in Brown et al. (2004) for Hawaiian reefs. During extensive studies in 1998, Brown et al. (2004) found that repeated sampling of conventional transects or quadrats showed unacceptably high variation unless fixed transects were established to allow precise repositioning. Statistical power to detect change in coral cover decreased dramatically when coral cover was greater than 20%. Longer transects (e.g., 25 m and 50 m) fared well in homogeneous substrates but shorter transects (e.g., 10 m) were more appropriate in heterogeneous habitats. Variability between observers analyzing the same data was low in comparison to other sources of error. Visual estimation techniques were cost effective but did not permit data archiving of digital images. Digital imaging had the highest initial monetary investment but yielded the largest quantity of data per unit of field effort. These results were used to establish the standard CRAMP monitoring sites that can detect less than a 10% change in coral cover with high statistical power (*P* > 0.80) using 50 points per frame, 20–30 frames per transect and 8–10 transects of 10 m in length per site. The power of this design increases over time with repeated surveys. In the CRAMP protocol, fixed photoquadrats with high precision are also used to address questions on recruitment, growth and mortality. The recent production of precise habitat maps along with introduction of the geographic positioning system (GPS) and availability of geographic information systems (GIS) has opened the possibility of using stratified random sampling of coral communities in Hawaiʻi as an alternative to fixed transects, but again this approach requires a large number of transects per habitat to detect change due to the inherently high variance of coral coverage data. An extensive summary and review of all aspects of coral reef monitoring (questions, techniques and design) has been compiled by Hill & Wilkinson (2004).

## Conclusions

Over the past three decades numerous underwater survey methods have been used to estimate coral cover throughout the Hawaiian Archipelago. These methods vary in technical complexity, the cost of equipment required, the amount of time required to conduct the survey, and the time needed to analyze images and record data. Some methods are more appropriate for rapid low-cost surveys while others are more suitable for intensive detailed analyses of cover that require digital images of coral community structure which are analyzed in the laboratory and archived for future reference. The choice of method is dependent on the question being asked. Some studies only involve measures of coral cover while others involve simultaneous measurements of factors such as coral, algae, fishes, invertebrates and rugosity. In these cases, the operational compatibility of the coral census method with other assessments influences method selection.

Results indicate that each of the nine methods tested in this investigation is appropriate for measuring coral cover and all methods provide a good first estimate for the general characterization of coral cover on a reef. However, it has long been known that a large number of short “quick and dirty” transects will produce a better estimate of coral cover in a given habitat than a few highly detailed longer transects (Kinzie & Snider, 1978; Brown et al., 2004). A method that yields more transects with the same amount of effort will generally be superior in quantifying coral cover in a given habitat.

The nine methods evaluated in this study were originally developed in response to the needs of specific research projects. Some of the factors that led to the development of these methods include the question being asked (intensive or extensive surveys), field and laboratory processing time restrictions, costs required, observer field experience, utility of sampling regimes under various field conditions, and archival capabilities. Results of this investigation indicate that investigators who initially developed and implemented each method had a strong “intuitive” understanding of how to develop a reasonable method that accurately measures coral cover, among their other survey needs. It is of great significance to managers, researchers, and modelers to know that all of these methods yield similar coral cover estimates since these methods that have been widely used in the past will undoubtedly continue to be used in the future. This study only compared transect methods and did not address the question of long-term monitoring design which has been considered elsewhere.

Beenaerts & Vanden Berghe (2005) compared a line transect method (LTM), line intercept method (LIT) and a point intercept transect (PIT). The LTM is similar to the LIT but follows the contours of the coral and substrate. They concluded that results from the three methods were virtually indistinguishable.” We can describe this relationship as LTM = LIT = PIT. Nadon & Stirling (2006) searched the recent literature and found that of the three most commonly used methods, the belt transect (BT) method was used twice as much as the LTM method and 2.5 times as often as the PIT method. The correlation coefficients for three ecological parameters were calculated for the three possible pairs of methods, and the accumulation curves plotted for each of the parameters using number of transects as the independent variable. The authors conclude that results from the three methods were virtually indistinguishable (i.e., BT = LTM = PIT). When the parameters were plotted using measuring time on the *x* axis, the curves for the PIT method converged twice as fast as those for LTM, while BT time was intermediate. It is suggested that the PIT method might be most suitable for assessing coral cover, richness and diversity where time and effort are significant constraints. Results of their study indicates that the least used method (PIT) was the most cost-effective means of measuring coral cover while being at least as precise and accurate as the other two methods. Combining the conclusions of both studies leads to the logic that LTM = LIT = PIT = BT. Recent improvements in digital imaging allows for inexpensive and rapid photo documentation and archiving with subsequent analysis of the images on computers in the comfort of the laboratory. This has become the method of choice in Hawaiʻi and other locations using the BT approach. Josephitis et al. (2012) compared the effectiveness and relative cost (processing time) of three image analysis techniques commonly used to assess coral/benthic cover and coral bleaching. Digital photographs along a BT, taken 1 m above the substrate at 1 m intervals along 16 transects (50 images per transect), were used to examine the extent of coral cover and bleaching within coral communities. Each image was evaluated by: (1) assessing habitat under six randomly placed points (‘point count’); (2) dividing images into 20 square blocks and recording the dominant item in each block (‘block’); and (3) visually estimating benthic cover and bleaching without reference to points or grids (‘visual’). Overall, there was a high degree of congruence between the commonly used techniques and there were no significant differences when comparing coral cover or the extent of bleaching. Similarly, there was no detectable difference in the precision of coral and bleaching estimates made using the three techniques. The comparison of the nine methods described in the present study support, expand and amplify the results of the previous investigations and further show the similarity of results from BT, LTM, LIT, PIT and visual estimate methods. The one caveat of concern is that line methods can yield a slightly higher coral cover, especially under conditions of high surge, because the line will tend to hang up on coral heads and bias the result. Use of a chain along a line transect or following contours adds a three dimensional aspect that will yield higher coral cover, especially in areas of high relief of living coral colonies. Inshore coral reefs in Hawaiʻi are characterized by heavy wave action that moves chain across the bottom. Therefore, the use of chain transects in Hawaiʻi has been discouraged by management agencies as well as scientists due to resulting coral breakage.

The results of this investigation lead us to the encouraging observation that in many applications coral cover measured by different methods can be legitimately combined. This conclusion has been supported directly by the success of recent modeling efforts based on a benthic cover database consisting of historical measurements taken in Hawaiʻi using the different methods and different observers over the past decade (Franklin, Jokiel & Donahue, 2013). These data were used to successfully develop and test species distribution models for dominant Hawaiian coral species.

## Supplemental Information

10.7717/peerj.954/supp-1 Supplemental Information 1 Methods Comparison data Excel worksheet with raw data information

## References

[ref-1] Aeby GS, Williams GJ, Franklin EC, Kenyon J, Cox EF, Coles SL, Work TM (2011). Patterns of coral disease across the Hawaiian Archipelago: relating disease to environment. PLoS ONE.

[ref-2] Beenaerts N, Vanden Berghe E (2005). Comparative study of three transect methods to assess coral cover, richness and diversity. Western Indian Ocean Journal of Marine Science.

[ref-3] Brown EK, Cox E, Jokiel PL, Rodgers SK, Smith WR, Tissot B, Coles SL, Hultquist J (2004). Development of benthic sam pling methods for the Coral Reef Assessment and Monitoring Program (CRAMP) in Hawaiʻi. Pacific Science.

[ref-4] Carleton JH, Done TJ (1995). Quantitative video sampling of coral reef benthos: large-scale application. Coral Reefs.

[ref-5] Coles SL (1984). Colonization of Hawaiian reef corals on new and denuded substrata in the vicinity of a Hawaiian power station. Coral Reefs.

[ref-6] Congalton R (1988). A comparison of sampling schemes used in generating error matrices for assessing the accuracy of maps generated from remotely sensed data. Photogrammetric Engineering and Remote Sensing.

[ref-7] Coyne MS, Battista TA, Anderson M, Waddell J, Smith WR, Jokiel PL, Kendell MS, Monaco ME (2003). NOAA technical memorandum NOS NCCOS CCMA 152. Benthic habitats of the main Hawaiian Islands.

[ref-8] Dethier MN, Graham ES, Cohen S, Tear LM (1993). Visual versus random-point percent cover estimations: ‘objective’ is not always better. Marine Ecological Progress Series.

[ref-9] Dollar SJ (1982). Wave stress and coral community structure in Hawaii. Coral Reefs.

[ref-10] Dollar SJ, Grigg RW (2004). Anthropogenic and natural stresses on selected coral reefs in Hawaiʻi: a multidecade synthesis of impact and recovery. Pacific Science.

[ref-11] Franklin EC, Jokiel PL, Donahue MJ (2013). Predictive modeling of coral distribution and abundance in the Hawaiian Islands. Marine Ecological Progress Series.

[ref-12] Green RH, Smith SR (1997). Sample program design and environmental impact assessment on coral reefs.

[ref-13] Hill J, Wilkinson C (2004). Methods for ecological monitoring of coral reefs.

[ref-14] Huse I, Løkkeborg S, Soldal AV (2000). Relative selectivity in trawl, longline and gillnet fisheries for cod and haddock—ICES. Journal of Marine Science.

[ref-15] Jokiel PL, Brown EK (1998). Coral baseline survey of Maalaea Harbor for light draft vessels, Island of Maui. Final Report for DACW83-96-P-0216.

[ref-16] Jokiel PL, Brown EK, Friedlander A, Rodgers KS, Smith WR (2004). Hawaii coral reef assessment and monitoring program: spatial patterns and temporal dynamics in reef coral communities. Pacific Science.

[ref-17] Jokiel PL, Coles SL (1974). Effects of heated effluent on hermatypic corals at Kahe Point, Oahu. Pacific Science.

[ref-18] Jokiel PL, Cox EF, Crosby MP (1993). An evaluation of the nearshore coral reef resources of Kahoolawe, Hawaii. Final Report for Co-operative Agreement NA27OM0327.

[ref-19] Jokiel PL, Tyler WA (1992). Distribution of stony corals in Johnston Atoll lagoon.

[ref-20] Jokiel PL, Maragos JE, Smith SV, Henderson RS (1978). Reef corals of Canton Atoll: II. Local distribution. Phoenix Islands Report I. An environmental survey of canton atoll lagoon 1973.

[ref-21] Josephitis E, Wilson S, Moore JAY, Field S (2012). Comparison of three digital image analysis techniques for assessment of coral cover and bleaching. Conservation Science Western Australia.

[ref-22] Kenchington RA, Stoddart DR, Johannes RE (1978). Visual surveys of large areas of coral reefs. Coral reefs: research methods.

[ref-23] Kenyon JC, Aeby GS, Brainard RE, Chojnacki JD, Dunlap MJ, Wilkinson CB (2006b). Mass coral bleaching on high-latitude reefs in the Hawaiian Archipelago.

[ref-24] Kenyon JC, Brainard RE, Hoeke RK, Parrish FA, Wilkinson CB, Somerton DA, Glendhill CT (2005). Towed diver surveys, a method for mesoscale spatial assessment of benthic reef habitat: a case study at Midway Atoll in the Hawaiian Archipelago. Report of the national marine fisheries workshop on underwater video analysis, NOAA Technical Memorandum NMFS-F/SPO-68.

[ref-25] Kenyon JC, Vroom P, Page KN, Dunlap MJ, Wilkinson CB, Aeby GS (2006a). Community structure of hermatypic corals at French Frigate Shoals, Northwestern Hawaiian Islands: capacity for resistance and resilience to selective stressors. Pacific Science.

[ref-26] Kinzie RA, Snider RH, Stoddart DR, Johannes RE (1978). A simulation study of coral reef survey methods. Coral reefs: research methods.

[ref-27] Loya Y (1972). Community structure and species diversity of hermatypic corals at Eilat, Red Sea. Marine Biology.

[ref-28] Maragos JE (1972). A study of the ecology of Hawaiian reef corals. Ph.D. thesis.

[ref-29] Maragos JE, Grover-Dunsmore (1999). Proceedings of the Hawaiʻi coral reef monitoring workshop.

[ref-30] Maragos JE, Jokiel PL (1986). Reef Corals of Johnston Atoll: One of the world’s most isolated reefs. Coral Reefs.

[ref-31] Miller TJ (2013). A comparison of hierarchical models for relative catch efficiency based on paired-gear data for US Northwest Atlantic fish stocks. Canadian Journal of Fisheries and Aquatic Sciences.

[ref-32] Monaco ME, Christensen JD, Friedlander AM, Kendall MS, Caldow C (2005). Quantifying habitat utilization patterns of US Caribbean and Hawaii reef fish to define marine protected area boundaries: the coupling of GIS and ecology.

[ref-33] Nadon MO, Stirling G (2006). Field and simulation analyses of visual methods for sampling coral cover. Coral Reefs.

[ref-34] NOAA (2005). Detailed Methodology—NOAA’s Biogeography Program. http://biogeo.nos.noaa.gov/projects/reef_fish/protocols.shtml.

[ref-35] Obura DO (2014). Coral reef monitoring manual.

[ref-36] Parrish FA, Polovina JJ (1994). Habitat thresholds and bottlenecks in production of the spiny lobster (*Panulirus marginatus*) in the Northwestern Hawaiian Islands. Bulletin of Marine Science.

[ref-37] Porter JW (1972). Patterns of species diversity in Caribbean reef corals. Ecology.

[ref-38] Reed AS (1980). Sampling and transecting techniques on tropical reef substrates.

[ref-39] Rodgers KS, Jokiel PL, Brown EK, Hau S, Sparks R (2015). Over a decade of change in spatial and temporal dynamics of Hawaiian coral reef communities. Pacific Science.

[ref-40] Rogers CS (1999). Sampling may be hazardous to your reef monitoring program.

